# Assessing potential shared genetic aetiology between body mass index and sleep duration in 142,209 individuals

**DOI:** 10.1002/gepi.22174

**Published:** 2018-11-26

**Authors:** Victoria Garfield, Ghazaleh Fatemifar, Caroline Dale, Melissa Smart, Yanchun Bao, Clare H. Llewellyn, Andrew Steptoe, Delilah Zabaneh, Meena Kumari

**Affiliations:** ^1^ Department of Behavioural Sciences & Health University College London London UK; ^2^ Department of Population Science & Experimental Medicine Institute of Cardiovascular Science University College London London UK; ^3^ Institute of Health Informatics University College London London UK; ^4^ Institute for Social and Economic Research University of Essex Colchester UK; ^5^ Social, Genetic & Developmental Psychiatry Centre, Institute of Psychiatry, Psychology & Neuroscience King's College London London UK; ^6^ Department of Epidemiology & Public Health University College London London UK

**Keywords:** body mass index, genetic correlation, polygenic risk score, sleep duration

## Abstract

Observational studies find an association between increased body mass index (BMI) and short self‐reported sleep duration in adults. However, the underlying biological mechanisms that underpin these associations are unclear. Recent findings from the UK Biobank suggest a weak genetic correlation between BMI and self‐reported sleep duration. However, the potential shared genetic aetiology between these traits has not been examined using a comprehensive approach. To investigate this, we created a polygenic risk score (PRS) of BMI and examined its association with self‐reported sleep duration in a combination of individual participant data and summary‐level data, with a total sample size of 142,209 individuals. Although we observed a nonsignificant genetic correlation between BMI and sleep duration, using LD score regression (*r*_g_ = −0.067 [*SE* = 0.039], *P* = 0.092) we found that a PRS of BMI is associated with a decrease in sleep duration (unstandardized coefficient = −1.75 min [*SE* = 0.67], *P* = 6.13 × 10^−7^), but explained only 0.02% of the variance in sleep duration. Our findings suggest that BMI and self‐reported sleep duration possess a small amount of shared genetic aetiology and other mechanisms must underpin these associations.

## 1 INTRODUCTION

A large number of studies find an association between body mass index (BMI) and self‐reported sleep duration in adults such that a higher BMI is associated with shorter duration of sleep (Cappuccio et al., 2008; Chaput, Després, Bouchard, & Tremblay, 2008; Gangwisch, Malaspina, Boden‐Albala, & Heymsfield, 2005; Garfield, Llewellyn, Steptoe, & Kumari, 2017; Hasler et al., 2004; Mezick, Wing, & McCaffery, 2014; Ogilvie et al., 2016; Patel & Hu, 2008; Patel, Malhotra, White, Gottlieb, & Hu, 2006). While the association between BMI and sleep duration may be bidirectional in nature (Vgontzas, Bixler, & Basta, 2010), with shorter sleep preceding weight gain (Chaput et al., 2008; Gildner, Liebert, Kowal, Chatterji, & Josh Snodgrass, 2014; Lauderdale et al., 2009), recent evidence suggests that the direction of association may also be from increased BMI to reduced sleep (Garfield et al., 2017; Stranges et al., 2008).

A recent study analysed genome‐wide data from ~120,000 UK Biobank (UKB) individuals to investigate whether there is a genetic correlation between BMI and sleep duration (Jones et al., 2016). They found a genetic correlation between BMI and oversleeping (defined as >8 vs. 7–8 hours): *r*_g_ = 0.097, *P* = 0.04; BMI and undersleeping (defined as <7 vs. 7–8 hours): *r*_g_ = 0.147, *P* = 1 × 10^−5^, but not BMI and continuous sleep duration (*r*_g_ = −0.05, *P* = 0.11). Interestingly, finding that BMI and undersleeping are genetically correlated supports the epidemiological literature which suggests an association between less sleep and higher BMI (Cappuccio et al., 2008; Chaput et al., 2008; Gangwisch et al., 2005; Garfield et al., 2017; Hasler et al., 2004; Mezick et al., 2014; Ogilvie et al., 2016; Patel & Hu, 2008; Patel et al., 2006). However, the biological underpinning of this association is unclear. Therefore, further work is required to examine whether BMI and self‐reported sleep duration possess underlying shared genetic aetiology, which can be carried out using polygenic risk scores (PRS). This method can detect whether a common genetic basis exists between related traits or diseases and could provide prediction of an individuals’ genetic risk for a particular disease or outcome (Dudbridge, 2013). Unlike an *r*_g_ obtained using molecular methods, such as LD Score (LDSC) regression, PRS provides a more refined approach to the investigation of shared genetic factors between two traits. PRS methods can now take into account correlations between single nucleotide polymorphism (SNPs) (known as linkage disequilibrium [LD]) and thin them accordingly, which means that we can work with a high‐resolution PRS for a particular trait, rather than merely genome‐wide association study (GWAS) significant SNPs, or all SNPs from GWAS summary statistics.

Thus, the present study aimed to perform the first thorough investigation of the shared genetic predictors between BMI and self‐reported sleep duration. We used PRSs to examine whether BMI and sleep duration have a common genetic basis in our data. We analysed a combination of individual participant data (IPD) and summary‐level data in up to 142,209 individuals (Jones et al., 2016) by including two datasets in addition to UKB we aimed to increase our statistical power, compared to the previous study. Our aims were threefold: (a) To perform GWAS analyses of self‐reported sleep duration in two population‐based studies, as this formed the basis of our outcome data, (b) to examine the association of a BMI PRS with sleep duration, and (c) to estimate the genetic correlation (*r*_g_) between BMI and sleep duration in our studies using LD score regression (Bulik‐Sullivan et al., 2015).

## 2 MATERIALS AND METHODS

### 2.1 Sample(s)

Our study included summary‐level data from 127,573 UKB participants (Jones et al., 2016), as well as IPD from two population studies: The English Longitudinal Study of Ageing (ELSA) and Understanding Society: UK Household Longitudinal Study (UKHLS).

ELSA is an on‐going national panel study of health and ageing, which started in 2002–2003 (wave 1). Data have been collected from respondents at waves 2 (2004–2005), 3 (2006–2007), 4 (2008–2009), 5 (2010–2011) and 6 (2012–2013), and comprise a nationally representative sample of English household residents 50 years and over. A more detailed account of ELSA can be found elsewhere (Steptoe, Breeze, Banks, & Nazroo, 2013). ELSA was granted ethical approval by the London Multicentre Research Ethics Committee (MREC 01/2/91) and all respondents provide informed consent at every wave. During the waves 2 and 4 nurse visits blood samples were taken from 5,662 and 1,945 participants, respectively, who provided informed consent to have DNA extracted for genotyping.

UKHLS is a household panel study, which covered approximately 32,000 households across the United Kingdom at wave 1 (Knies, 2016). UKHLS received ethical approval from the University of Essex Ethics Committee and the nurse data collection by the National Research Ethics Service (10/H0604/2). During 2010–2012 (wave 2 or 3) approximately 20,000 respondents aged 16+ were invited to take part in a Nurse Health Assessment interview. In ~13,000 of these respondents a blood sample was taken and DNA was extracted for genotyping from ~10,000 individuals, once informed consent was obtained.

In both ELSA and UKHLS, all methods were carried out in accordance with approved guidelines and regulations.

Summary statistics from the recent UKB GWAS of sleep duration (Jones et al., 2016) were also used in our genetic analyses. These authors performed linear mixed modelling

Table 1 presents the number of individuals included in each type of analysis. For PRS and genetic correlation analyses we had a total sample size of 142,209 (ELSA + UKHLS + UKB sleep duration GWAS individuals) and for phenotypic analyses we used 12,107 individuals (ELSA + UKHLS IPD).

**Table 1:** Details of samples included in this study, with respective *N*s for different analyses

Study	Type of data	Number of individuals in GWAS	Number of individuals in phenotypic analyses
ELSA	IPD	6,028	5,296
UKHLS	IPD	8,608	6,811
UKB	Summary^a^	127,573	N/A
Total *N*	N/A	142,209	12,107

*Note*. ELSA: English Longitudinal Study of Ageing; IPD: individual participant data; UKB: UK Biobank; UKHLS: UK Household Longitudinal Study.

^a^ Summary statistics from sleep duration GWAS by Jones et al. (2016) downloaded from http://www.t2diabetesgenes.org/data/

### 2.2 Measures

#### 2.2.1 BMI and sleep duration

BMI was calculated (weight in kg/height in metres squared) from height and weight measured by a nurse using standardized protocols in both ELSA and UKHLS. Sleep duration was self‐reported across both studies. Specifically, ELSA respondents were asked: “How many hours of sleep do you have on an average week night?” UKHLS participants were asked: “How many hours of actual sleep did you usually get at night during the last month? This may be different than the actual number of hours you spent in bed.”

### 2.3 Genotyping

ELSA and UKHLS were genotyped using the Illumina Omni 2.5M and HumanCoreExome arrays, respectively. Standard quality control (QC) procedures for genetic data were carried out in both ELSA and UKHLS. In ELSA, both SNP and individual missingness were set to 5%. In UKHLS, individual and SNP missingness were set to 2%, duplicate individuals were removed, as were SNPs with a Hardy Weinberg equilibrium *P* < 1 × 10^−4^. An identity by descent (IBD) cut‐off of 0.025 was used, which excludes individuals who are related up to third or fourth cousins. Therefore, of the original sample (*n* = 9,994) who were genotyped and passed QC, IBD excluded *n* = 1,001 individuals (one per related pair). This method selects one individual per pair to keep/exclude at random.

In both studies individuals who reported non‐White ethnicity were removed; sex discrepancies were also corrected. We also removed 109 related individuals in ELSA and 1,001 (as described above) in UKHLS before performing the analyses. Details of genotyping in the UKB study are described elsewhere (Wain et al., 2015). Both studies were imputed to the European component of 1,000 genomes.

### 2.4 Statistical analyses

#### 2.4.1 Phenotypic analyses

In studies for which we had IPD (ELSA and UKHLS), phenotypic association analyses were performed using linear regressions, with adjustments for age and sex. These results were subsequently combined in a fixed‐effects meta‐analysis to obtain an overall estimate for both studies. Heterogeneity between studies was assessed by means of Cochran's Q and *I* ^2^.

#### 2.4.2 Genetic analyses

##### 2.4.2.1 Genome‐wide analyses of sleep duration

As mentioned previously, summary statistics from the latest published sleep duration GWAS (Jones et al., 2016) were included in our analyses. This GWAS was adjusted for age, sex and study centre and these authors used linear mixed modelling in BOLT‐LMM (Loh et al., 2015), which is able to take into account potential relatedness between individuals.

A GWAS of sleep duration was conducted with 1,000 genomes imputed data set from ELSA and UKHLS using linear regressions, adjusted for age, sex and 10 principal components in the R package (version 3.3.2), snpStats (Clayton, 2014). As UKHLS is a household study, there is a greater proportion of close relatives, thus we used an IDB cut‐off of 0.025, which excludes individuals who are up to fourth‐degree cousins and retains one individual per pair.

Results from ELSA, UKB and UKHLS studies were meta‐analysed using inverse‐variance fixed effects in METAL, to obtain an overall estimate per SNP.

#### 2.4.3 PRSs of BMI

As the main aim of our analyses was to identify whether BMI and self‐reported sleep duration possess shared common genetic factors, we sought to use genome‐wide summary statistics from published GWA studies of BMI and sleep duration, to have a very large sample size. PRSs were created using an additive model and by choosing a broad *P*‐value threshold inclusion range (described below) and at each of these thresholds a PRS was produced that comprised all possible SNPs that overlap between BMI and sleep duration.

Summary statistics were downloaded from a recently published large‐scale consortium meta‐GWAS of BMI (Locke et al., 2015) from up to 339,224 individuals, and these β coefficients were used as external weights in our PRSs. Our outcome data were summary statistics from the meta‐GWAS of sleep duration described above (Jones et al., 2016). There should be no overlap between the exposure and outcome data, in terms of the samples analysed, which our data complied with. PRS analyses were performed using PRSice, version 1.25 (Euesden, Lewis, & O'Reilly, 2015).

A total of nine PRSs at *P*‐value thresholds of 1.0, 0.5, 0.4, 0.3, 0.2, 0.1, 0.05, 0.01 and 0.001 were created (Table 3). At each threshold SNPs were clumped by LD using a cut‐off of *r* ^2^ = 0.1 and a window of 250 kb, to ensure that independent SNPs were included in each PRS.

#### 2.4.4 Genetic correlation (*r*_g_) of BMI and sleep duration

Before performing the *r*_g_ analysis a set of QC procedures are carried out automatically when summary statistics are submitted to LD Hub; this process is described in detail in a recent publication (Zheng et al., 2017). LD score regression was used to estimate the genetic correlation (*r*_g_) between BMI and sleep duration (Bulik‐Sullivan et al., 2015) using LD Hub (Zheng et al., 2017). Summary statistics from GWA analyses of sleep duration in ELSA, UKHLS and UKB were uploaded to examine the underlying *r*_g_ of BMI with sleep duration in our whole sample.

## 3 RESULTS

### 3.1 Sample characteristics

Table 2 shows sample characteristics for individuals from ELSA and UKHLS included in the phenotypic analyses. Mean sleep duration and BMI was similar across both studies.

**Table 2:** Participant characteristics for individual participant data studies (*N* = 12,107)

Study	Mean sleep duration (hours; *SD*)	Mean BMI (kg/m^2^; *SD*)	Mean age (*SD*)	Female (%)
ELSA (5,296)	6.86 (1.27)	28.14 (5.11)	66.7 (9.16)	54.19
UKHLS (6,811)	6.63 (1.29)	28.01 (5.05)	52.76 (15.98)	56.03
Both studies (12,107)	6.74 (1.28)	28.07 (5.08)	59.73 (12.57)	56.57

*Note*. BMI: body mass index; ELSA: English Longitudinal Study of Ageing; UKHLS: UK Household Longitudinal Study.

### 3.2 Phenotypic analyses of BMI and sleep duration

**Figure 1:**
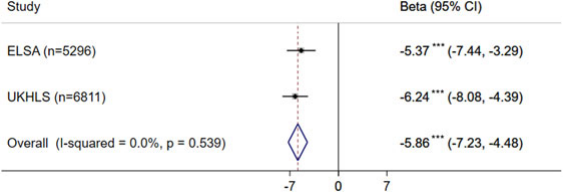
Phenotypic association between (standardised) body mass index and sleep duration in English Longitudinal Study of Ageing (ELSA) and UK Household Longitudinal Study (UKHLS; *N* = 12,107). 95% CI: 95% confidence interval, ***P < 0.001

The estimates presented in Figure 1 are adjusted for age and sex, and depict a negative association between BMI and self‐reported sleep duration, such that for every SD increase in BMI (kg/m^2^) sleep duration decreases by 5 and 6 minutes in ELSA and UKHLS, respectively. Our overall estimate showed that for every SD increase in BMI (5.08 kg/m^2^) sleep duration decreased by 5.86 minutes. Cochran's *Q* and *I* ^2^ revealed that there were no issues with heterogeneity between the two studies (Cochran's *Q*, *P* = 0.70, *I* ^2^ = 0).

### 3.3 Associations between PRSs of BMI with sleep duration

Of the nine PRSs, a PRS of BMI at a *P*‐value inclusion threshold of 0.01 explained the highest proportion of the variance in sleep duration (0.02%). This PRS was negatively associated with sleep duration (*B* = −1.75, *P* = 6.13 × 10^−7^; Table 3).

**Table 3:** Polygenic risk score analyses of body mass index and sleep duration in 142,209 individuals after clumping SNPs by linkage disequilibrium (LD)

*P*‐value threshold	No. of SNPs in model	Coefficient (*SE*)	*P* value	(Pseudo) *R* ^2^
1.0	54,505	−0.62 (0.18)	0.0003	6.06 × 10^−5^
0.5	36,688	−0.64 (0.18)	0.0003	6.94 × 10^−5^
0.4	31,254	−0.59 (0.19)	0.0008	7.49 × 10^−5^
0.3	25,196	−0.67 (0.20)	0.0003	8.16 × 10^−5^
0.2	18,195	−0.62 (0.21)	0.002	8.22 × 10^−5^
0.1	10,477	−0.80 (0.24)	0.0005	8.44 × 10^−5^
0.05	6,006	−1.33 (0.29)	2.36 × 10^−6^	0.0001
0.01^a^	2,024	−1.75 (0.67)	6.13 × 10^−7^	0.0002
0.001	536	−2.07 (0.43)	0.004	4.83 × 10^−5^

*Note*. LD clumping parameters are *r* ^2^ = 0.1 and 250 kb. Coefficient indicates unstandardized coefficient in minutes of sleep duration.

^a^ Best threshold with 0.02% of the variance in sleep duration explained by this PRS.

### 3.4 Genetic correlation (*r*_g_) of BMI and sleep duration

The genetic correlation between BMI and sleep duration using LD score regression was not significant, *r*_g_ = −0.067 (*SE* = 0.039), *P* = 0.092, yet it was consistent with the negative direction of effect observed in our results using PRSs.

## 4 DISCUSSION

In the study reported here, we performed a comprehensive set of analyses to investigate potential shared genetic aetiology between BMI and sleep duration, by means of PRSs and genetic correlations. We exploited summary‐level data from the latest UKB GWAS of sleep duration (Jones et al., 2016), alongside two studies for which we had individual‐level data, to form a combined sample of 142,209 individuals.

Phenotypic findings from our meta‐analysis of 12,107 individuals are consistent with recently published meta‐analyses of a negative association between BMI and sleep duration in adults (Cappuccio et al., 2008; Patel & Hu, 2008). Our genetic findings suggest that the best‐fit BMI PRS was significantly negatively associated with sleep duration, but explained only 0.02% of its variance. We also found no significant genetic correlation between BMI and sleep duration using LD score regression, a finding that supports recent research which found a similar genetic correlation in terms of strength and direction, *r*_g_ = −0.05 (Jones et al., 2016).

To find the highest resolution BMI PRS and examine its association with sleep duration, we constructed nine distinct PRSs in an attempt to find the best one. The optimal PRS created was negatively associated with sleep duration. However, this PRS accounted for only 0.02% of the variance in self‐reported sleep duration. While perhaps BMI and sleep duration in fact, do not possess shared genetic aetiology, it is also possible that the sleep duration phenotype is error‐prone which made it more difficult to detect the true common aetiology.

Moreover, our estimate of −1.75 minutes is less than half the size of the phenotypic effect we found. However, our phenotypic analyses were not performed on the entire sample of 142,209, but only in ELSA and UKHLS and thus may not be completely comparable with the effect size from our PRS analyses. It is also important to note that we would expect the genetic effect from the PRS analyses to be smaller than the phenotypic effect. Although the exact biological mechanisms for the association between our BMI PRS and sleep duration remain largely elusive, we can begin to speculate on some of the potential underlying pathways. The well‐characterised fat‐mass and obesity‐associated (FTO) gene is of interest here, as it is expressed in the hypothalamus (Locke et al., 2015) and neurons in the ventrolateral preoptic nucleus are instrumental in promoting sleep, by shutting off other arousal centres in the brain (Saper, Scammell, & Lu, 2005). Specifically, rs1558902, an intron in the FTO gene was included in our best‐fit PRS and its association effect with sleep duration in our meta‐GWAS of all three studies was −0.68 minutes. Thus, although this effect was not genome‐wide significant (*P* = 0.003) it is consistent with the expected direction of effect, such that higher genetic risk of obesity associates with shorter sleep. Future research could perform downstream functional analysis to investigate whether any well‐established BMI genetic variants are on relevant causal pathways for sleep duration.

This study has several strengths. For the first time, polygenic risk scoring techniques were applied to investigate the potential underlying genetic overlap of BMI with self‐reported sleep duration. We used a large sample, by analysing both IPD and summary‐level data; we weighted our PRS externally using published GWAS estimates for BMI (Locke et al., 2015), which reduced bias in the results, as none of the studies we analysed contributed to this GWAS. However, we also acknowledge certain limitations of our study. We analysed self‐reported sleep duration, which might suffer from measurement error and bias (Van Den Berg, Van Rooij et al., 2008). Our findings are conducted with data from White/European individuals and may therefore not be applicable to other ethnic groups. Our data were cross‐sectional; therefore, future research should investigate the association of BMI PRSs with sleep duration over time. We did not have access to the individual data for UKB, thus we were not able to include this sample in our phenotypic analyses, nor were we able to include covariates in our PRS analyses. Finally, the sleep duration question was slightly different in each of the three studies analysed here. The UKB study asked respondents to report the average number of hours slept in a 24‐hour period, while ELSA specifically asked to provide the average number of hours slept on weeknights only, and UKHLS asked respondents for the hours of actual sleep during the last month, while informing them that this may be different from the total hours spent in bed.

Some important future directions for this study should also be considered. It is essential to perform similar analyses in future work using objectively measured sleep duration, as subjective measures are prone to error and bias (Van Den Berg et al., 2008), and are correlated only moderately with objective measurements (Arora, Broglia, Pushpakumar, Lodhi, & Taheri, 2013; Van Den Berg et al., 2008). Future studies should investigate the possibility of shared genetic aetiology of BMI with other sleep dimensions, such as pattern, bedtime, quality and disturbance. This is of interest, as evidence suggests that each sleep phenotype may have effects that are independent of each other (Andretic, Franken, & Tafti, 2008). Findings from the present study require replication in distinct age groups. Although individuals’ DNA remains intact throughout their lives, changes in gene expression can occur and are linked to particular disease states and cellular responses (de Magalhães, Curado, & Church, 2009). Evidence also suggests that genetic influences on BMI vary considerably with age, even in childhood (Llewellyn, Trzaskowski, Plomin, & Wardle, 2014).

In summary, this study investigated shared underlying genetic aetiology between BMI and self‐reported sleep duration. We found that although a PRS of BMI was negatively associated with sleep duration, it accounted for a very small proportion of its variance. However, these findings warrant replication in other age and ethnic groups, with other sleep phenotypes and where possible, employing objective measures of sleep.

## AUTHOR CONTRIBUTIONS

V. G. conceived the study idea, performed the analyses, and wrote the manuscript. M. K. and D. Z. revised the manuscript for intellectual content. G. F. and D. Z. led the quality control of the ELSA genotype data. M. S. and Y. B. led the preparation of relevant phenotypic data for UKHLS analyses. M. K., D. Z., C. D., G. F., M. S., Y. B., C. H. L., and A. S. provided valuable comments and scientific insights to the manuscript. All authors approved the final manuscript.

## AVAILABILITY OF DATA

To access data from either ELSA or UKHLS visit the Managing Ethico‐social, Technical and Administrative issues in Data ACcess (METADAC) website https://www.metadac.ac.uk. The UK Biobank sleep duration GWAS summary statistics used in this paper can be accessed at: http://www.t2diabetesgenes.org/data/.

## CONFLICTS OF INTEREST

The authors declare that there are no conflicts of interest.
